# Analysis of Applicator Insertion Related Acute Side Effects for Cervical Cancer Treated With Brachytherapy

**DOI:** 10.3389/fonc.2021.677052

**Published:** 2021-06-07

**Authors:** Jiajun Chen, Ning Zhang, Ying Liu, Dongmei Han, Zhuang Mao, Wei Yang, Guanghui Cheng

**Affiliations:** Department of Radiation Oncology, China-Japan Union Hospital of Jilin University, Changchun, China

**Keywords:** cervical cancer, acute side effects, procedure, time, brachytherapy

## Abstract

**Purpose:**

To report applicator insertion-related acute side effects during brachytherapy (BT) procedure for cervical cancer patients.

**Materials and Methods:**

Between November 2017 and December 2019, 407 BT fractions were performed in 125 patients with locally advanced cervical cancer. Acute side effects recorded comprised anesthesia-related side effects, mechanical-related side effects and infection, whose frequency and degree were recorded. Pain was assessed using numeric rating scale; vaginal bleeding volume was counted by weighing gauze pieces used in packing. The BT procedure comprised eight stages: anesthesia, applicator insertion, image acquisition, transport, waiting for treatment, dose delivery, applicator removal, and removed which denoted 0.5–12.0 h period after removal, with time of each stage recorded. Factors influencing acute side effects were assessed by Spearman correlation and Mann–Whitney U test.

**Results:**

The most common acute side effect was pain, followed by vaginal bleeding. The mean scores for pain were highest during removal time, 4.9 ± 1.6 points. The mean vaginal bleeding volume was 44.4 ml during removal time. Mean total procedure time was 218.8 (175–336) min, having positive relationship with frequency of acute side effects. The total procedure time with acute side effects was longer than that without acute side effects. The longest procedure time was waiting time, 113.0 (91.0–132.0) min. More needles generated higher pain scores and larger volume of vaginal bleeding.

**Conclusion:**

Pain and vaginal bleeding were the most common acute side effects, especially during removal time, which physicians should focus on. Shortening patients’ waiting time helps to reduce the total procedure time, thus, reduce acute side effects. While meeting dose requirement, less needles are helpful to reduce acute side effects.

## Introduction

Cervical cancer is the fourth leading cause of cancer-related deaths in females worldwide (1), with 85% of cases reportedly occurring in developing countries (2). External beam radiotherapy (EBRT), combined with concurrent platinum-based chemotherapy followed by brachytherapy (BT), is the standard treatment for locally advanced cervical cancer (2–4). Brachytherapy has evolved to become an essential component of modern oncologic treatment for many locally advanced cervical cancers (5). BT comprises intracavitary brachytherapy (ICBT), interstitial brachytherapy (ISBT), and combined intracavitary/interstitial brachytherapy (IC/ISBT), which is based on ICBT that entails addition of needles to cover target area (4, 6). Currently, most physicians pay much more attention to late radiation morbidity after brachytherapy, rather than acute morbidity which may prolong overall treatment time (OTT) of radiotherapy and decrease the local control rates (7). Besides, complete and orderly brachytherapy is now guaranteed, and the efficiency of the process has also improved through having standard treatment procedure (8). However, few studies have comprehensively described the acute side effects during BT procedure for cervical cancer patients. The current study, therefore, sought to report applicator insertion-related acute side effects during BT procedure for cervical cancer patients by summarizing relevant clinical data at our department.

## Materials And Methods

### Patients

Between November 2017 and December 2019, 407 brachytherapeutic applications were performed at our department in 125 patients with biopsy proven cervical carcinoma. The mean age of the patients was 54 years (range 30–77 years). According to International Federation of Gynecology and Obstetrics (FIGO), 57 (45.6%) cases were present in stage IIB, 34 (27.2%), 17 (13.6%), eight (6.4%), five (4.0%), and two (1.6%) patients were diagnosed in stage IIIB, IIA2, IIIA, IVA, and IB2, and stages IIA1 (0.8%) and IVB (0.8%) were both represented in one patient (**Table 1**).

**Table 1:** Patient characteristics.

Characteristic	Value
Average age (years) (range)	54 (30–77)
BMI (range)	23 (17–33)
Chemotherapy, n (%)	
Concurrent chemotherapy	68 (54.4)
New adjuvant chemotherapy	23 (18.4)
Histology, n (%)	
Squamous cell carcinoma	119 (95.2)
adenocarcinoma	6 (4.8)
Tw_ICBT_ (cm) (SD)	3.9 (0.7)
Tw_IC/ISBT_ (cm) (SD)	
Tw_U_	5.3 (1.0)
Tw_R_	4.9 (0.7)
Tw_M_	4.9 (0.8)
Tw_ISBT_ (cm) (SD)	7.0 (1.4)
FIGO, n (%)	
IB2	2 (1.6)
IIA1	1 (0.8)
IIA2	17 (13.6)
IIB	57 (45.6)
IIIA	8 (6.4)
IIIB	34 (27.2)
IVA	5 (4.0)
IVB	1 (0.8)

BMI, Body Mass Index; SD, Standard Deviations; Tw_ICBT_, Tumor width of ICBT; Tw_IC/ISBT_, Tumor width of IC/ISBT; Tw_U_, Tumor width of IC/ISBT with Utrecht applicator; Tw_R_, Tumor width of IC/ISBT with Ring applicator; Tw_M_, Tumor width of IC/ISBT with multi-channel applicator; Tw_ISBT_, Tumor width of ISBT with self-made applicator.

### Treatment Procedure

**Figure 1:**
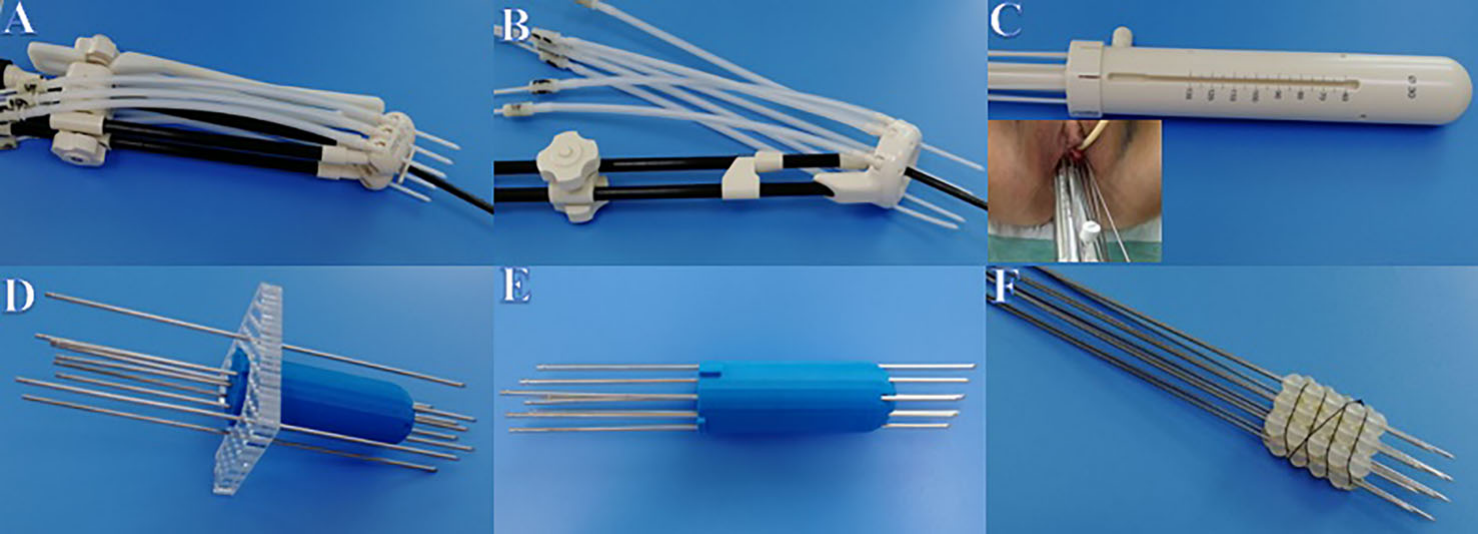
Applicators used in BT. **(A)** Utrecht applicator. **(B)** Ring applicator. **(C)** Multi-channel applicator. **(D)** Self-made template applicator. **(E)** Self-made 3D printing applicator. **(F)** Self-made silicone ball applicator.

All enrolled patients had previously received EBRT, with or without concurrent chemotherapy, followed by BT. Overall, EBRT was completed for 67 cases and 58 cases at our and other hospitals, respectively. The EBRT prescription to the pelvis was 45–50 Gray (Gy) in 25 fractions, and the BT prescription was 28 Gy over four fractions. Finally, patients undergoing ICBT and IC/ISBT used Utrecht, Ring, or Multi-channel applicators (United perineal insertion of needle), and those undergoing ISBT used self-made applicators (**Figure 1**).

### Brachytherapy Procedure

Preoperative preparation: MRI and gynecological examination were used to ascertain the location, size, shape, and invasion of tumor. Vaginal irrigation, enema, and catheterization were also performed on the patients before operation.

Operation: Patients were anesthetized before a radiation oncologist conducted preoperative disinfection in an operating room (OR). Thereafter, a transrectal ultrasound-guided insertion of the applicator and needles was performed under general anesthesia (GA). Finally, fixation of the applicator was completed using a self-made “T Type” fixing belt (9).

Waiting for patients wake up: These processes were performed by the anesthesiology team, radiation oncologists, a sonographer, and nurses.

Image acquisition: This procedure was performed by the MRI technicians in MRI room.

Waiting: Patients were transferred to the waiting room, where their conditions were monitored. At the same time, MRI images obtained in the MRI room were imported to the treatment planning system (TPS), where radiation oncologists contoured and physicists planned. The treatment plan was then reviewed and approved by the director. Finally, physicists re-checked the physics.

Dose delivery: The treatment plan was uploaded by the radiation oncologist and physicist, then the dose delivery was completed under real-time monitoring in the treatment room. Thereafter, the radiation oncologist carefully removed the applicator and needles then dealt with the resulting acute side effects after vaginal inspection in the waiting room.

The brachytherapy procedure was divided into eight stages: anesthesia, applicator insertion, image acquisition, patient transport, waiting for treatment, dose delivery, applicator removal, and removed. Among them, transport time included time spent on transferring patients from the OR to the MRI room and time spent on transferring patients from the MRI room to the waiting room. Waiting time contained importing, contouring, contour checking, planning, plan checking times. Removed denoted the period between 0.5 and 12.0 h after removal time.

### Data Collection

The total procedure time was the time between anesthesia and removal of applicator. The time taken for each procedure step was calculated for every patient. Acute side effects were assessed by a doctor during transport, waiting, removal, and removed periods. Hematocrit values from routine blood examinations before and after brachytherapy were recorded. Pain was estimated using the numeric rating scale (NRS) as painless (0 point), mild (1–3 points), moderate (4–6 points), severe (7–10 points), based on patient description. Vaginal bleeding volume was counted by weighing gauze pieces used in packing. Vaginal bleeding volume was recorded as 0–10 ml, 10–30 ml, 30–300 ml, and 300-ml. Tumor width was measured in MRI image of first brachytherapy.

### Statistical Analysis

Statistical analysis was performed using Statistical Programme for Social Sciences software (SPSS version 22.0) for Windows. Specifically, we collected process time and incidence of acute side effects during brachytherapy procedure. Continuous variables were described using means ± standard deviations (SDs), whereas categorical variables were described using rate or composition ratios. The depth of needle was described using median (interquartile range) and mode. Spearman correlation was used to analyze the relationship between the number of needles and incidence of acute side effects and relationship between the total procedure time and frequency of acute side effects; Mann–Whitney U test was used to compare total procedure time with and without acute side effects.

## Results

### Procedure Time

The mean total time was 218.8 (SD23.6; ranges 175–336) minutes (min). The mean BT fractions per day was 4 (range: 2–8). The mean associated time (min) for each stage was as follows. Anesthesia: 26.6 (SD10.8; ranges 5–68) min, insertion: 13.2 (SD8.1; ranges 3–53) min, transport time from OR to MRI room: 8.9 (SD 3.9; ranges 5–20) min, image acquisition: 25.7 (SD6.3; ranges 18–43) min, transport time from MRI room to the waiting room: 12.6 (SD 3.5; ranges 5–21) min, waiting time: 113.0 (SD12.6; ranges 91–132) min, dose delivery: 25.1 (SD12.4; ranges 2–84) min, removal: 15.2 (SD11.7; ranges 3–88) min **(** **Table 2**).

**Table 2:** The associated time required for each stage (min).

Procedure time	Mean	SD	Minimum	Maximum
Anesthesia	26.6	10.8	5	68
Insertion	13.2	8.1	3	53
T_OR-MR_	8.9	3.9	5	20
MRI	25.7	6.3	18	43
T_MR-WR_	12.6	3.5	5	21
Waiting	113.0	12.6	91	132
Importing	3.2	0.8	2	4
Contouring	27.7	12.8	10	58
Contour checking	24.0	6.1	15	36
Planning	30.7	12.8	15	64
Plan checking	27.3	8.2	16	41
Dose delivery	25.1	12.4	2	84
Removal	15.2	11.7	3	88
Total	218.8	23.6	175	336

T_OR-MR_, transport time from OR to MRI room; T_MR-WR_, transport time from MRI room to the waiting room.

### Applicators and Needles

The BT process was performed 407 times, with a mean number of needles 8.4 (SD4.3; ranges 1–28). Median depth of needles was 5 (interquartile ranges 3–5) cm, and the most frequent depth of needles also was 5 cm (1,736/2,922). The IC/ISBT process was performed 314 times, with 7.5 (SD3.7; ranges 1–17) needles. The ICBT process was performed 59 times. The ISBT process was performed 34 times, with 16.7 (SD4.9; ranges 10–28) needles (**Table 3**).

**Table 3:** Applicator and needles.

Types of applicators	Frequency of IC/ISBT	Frequency of ICBT	Numbers of needles	Applicator matches the needles		
				Mean ± SD	Minimum	Maximum
Utrecht applicator	207	29	1736	8.4 ± 4.0	1	17
Ring applicator	87	15	510	5.9 ± 2.4	2	12
Multi-channel applicator	20	15	107	5.4 ± 2.5	2	9
Total 1	314	59	2353	7.5 ± 3.7	1	17
Self-made applicator	34		569	16.7 ± 4.9	10	28
Total 2	348		2922	8.4 ± 4.3	1	28

### Acute Side Effects

Varied degrees of acute side effects were recorded in the 407 BT fractions. The mean number of acute side effects per fraction was 2.0 (SD 0.7; ranges 1–4). Pain was the most frequent acute side effect, with 407 fractions being recorded, followed by vaginal bleeding, 296 fractions. Vomiting, nausea, fever and dizziness were recorded in 36 fractions, 32 fractions, 25 fractions, and 12 fractions, respectively. Uterine perforation and hematuria were both recorded in five fractions. No other side effects such as deep vein thrombosis (DVT) was observed. More information was list in **Table 4**.

**Table 4:** Classification and proportion of acute side effects.

Classification	Types of side effects	Fractions (%)
Anesthesia related	Dizziness	12 (2.9)
	Nausea	32 (7.9)
	Vomiting	36 (8.8)
Operation related	Pain	407 (100)
	Vaginal bleeding	296 (72.7)
	Uterine perforation	5 (1.2)
	Hematuria	5 (1.2)
	Transplanted for organ damage in other areas	0
Infection	Fever	25 (6.1)
Others	Deep vein thrombosis	0
	Other serious complications	0

### Pain

Incidences of severe (rated 7–10) pain were identified in 75 BT fractions: during the removal time (65 fractions), during the waiting time (seven fractions), and transport time (three fractions). The mean NRS scores for pain were highest during removal time, 4.9 ± 1.6 points, followed by 2.1 ± 1.9 points, and 1.3 ± 1.5 points recorded during the periods of waiting and transport.

### Vaginal Bleeding

Vaginal bleeding was observed during removal time. Patients in 180 BT fractions had vaginal bleeding, with volume ranging 0–10 ml. Patients in 125 BT fractions, whose volume ranged 10–30 ml required vaginal packing or compression. Patients in 94 BT fractions, whose volume ranged 30–300 ml, required vaginal packing + compression + hemostatic drug. Patients in eight BT fractions, whose volume were more than 300 ml, required transfusion.

### Factors Influencing Acute Side Effects

Different methods of analysis were performed to assess factors influencing acute side effects (**Tables 5** and **6**). The number of needles and total procedure time were included in the analysis. Spearman correlation analysis revealed a positive association between the number of needles with the volume of vaginal bleeding and pain scores during removal time (*p* < 0.05) and a positive association between the total procedure time and frequency of acute side effects (*p* < 0.05). Besides, Mann–Whitney U test revealed the total procedure time with hematuria was longer than that without hematuria (*p* < 0.05).

**Table 5:** Factors influencing acute side effects.

Acute side effects	Mean ± SD	Z	*P*
Number of needles			
Volume of vaginal bleeding (ml)	44.4 ± 96.4	0.551	<0.001
Pain scores during removal (points)	4.9 ± 1.6	0.442	<0.001
Total treatment time (min)			
Frequency of acute side effects	2.0 ± 0.7	0.334	<0.001

**Table 6:** Total procedure time influencing acute side effects (min).

Acute side effects	Total procedure time (Mean ± SD)	Z	*P*
Hematuria			
Yes	252.60 ± 23.266	−2.850	0.004
No	218.40 ± 23.368		

## Discussion

MRI-based BT is a complex, time-constrained, and resource-intensive procedure, which can be challenging. Generally, the BT procedure comprises anesthesia, applicator insertion, image acquisition, patient transport, waiting for treatment, dose delivery, applicator removal and removed processes, whereas the acute side effects include anesthesia-related side effects and operative injury, pain, bleeding as well as infection (10–12). To the best of our knowledge, the present study is the first report describing acute side effects in BT procedure for cervical cancer. In the present study, we analyzed and summarized clinical data during brachytherapy for treatment of cervical cancer at our department to provide a reference for BT.

### The Brachytherapy Procedure Time

In the present study, our results showed that “waiting time” was the most time-consuming stage of the BT procedure. This might have led to prolonged braking in the waiting room, thereby predisposing patients to severe anxiety, claustrophobia, and pain (13, 14). These discomforts had previously been reported to cause patients to move and change the position of applicator and needles, thereby affecting the clinical outcomes (15, 16). Notably, shortening a patient’s waiting time played an important role in reducing the total procedure time and improving their comfort. The waiting time (113.0 min) in the present study was significantly longer than that spent in some institutions (17, 18). This could be attributed to the following reasons: Firstly, the radiation oncologists’ and physicists’ contouring, planning, checking speed were not fast during this period, which might result from caution. Assessment of brachytherapy time for treatment of cervical cancer patients by Kim et al. (18) revealed significantly shorter planning time in 2017 (63.2 min) than 2007–2008 (137.7 min). This benefited from a combined effect of technology and experience, resulting from acceleration of the speed of radiation oncologists’ target area drawing, physicists’ plan making and verification and the enhancement of proficiency following years of training. Secondly, a large number of patients received brachytherapy in one day, which led to queuing up at the radiation oncologists’ and physicists’ departments. Specifically, an average of four (range 2–8) patients underwent brachytherapy in one day at our department; more than two (range 1–4) reported by Kim et al. (18) whose average treatment time was 149.3 min. Finally, our department had only one high-dose-rate (HDR) remote after loading the brachytherapy machine, which made it impossible to simultaneously treat multiple patients. For reducing waiting time and improving BT procedure efficiency, we recommend controlling the number of patients undergoing the brachytherapy in a single day, improving proficiency of radiation oncologists and physicists and updating technology.

### Applicator and Needles

The statistics in **Tables 1** and **3** had proven that more needles were used with bigger tumor. Besides, Serban et al. (19) had summarized the average number of needles in IC/IS was 2.4 ± 1.2, 3.4 ± 1.2, 3.5 ± 1.8, and 4.8 ± 2.5 in Ring IC, Ovoids IC, Ovoids IC/IS, and Ring IC/IS centers, with tumor width diagnosis MR being 4.2 ± 1.4 cm, 4.5 ± 1.5 cm, 4.7 ± 1.4 cm, and 4.9 ± 1.4 cm, respectively. However, the relationship between volume of tumor and number of needles needs further study.

### Acute Side Effects

#### Anesthesia-Related Side Effects

Our results indicated that dizziness, nausea, and vomiting happened on 2.9% (12/407), 7.9% (32/407), and 8.8% (36/407) of all fractions, respectively, which was lower than 7%, 18%, and 14% for hypoxia, nausea, and vomiting reported by Watanabe et al. (20). The side effects were acceptable. According to an international survey of the Gynecologic Cancer Intergroup completed in 2009, 46% of anesthesia-assisted high-dose-rate (HDR) brachytherapy device insertions for cervical cancer involved general anesthesia, followed by 27% that used spinal anesthesia with intravenous (IV) conscious sedation (21). Currently, spinal anesthesia was found to have advantages over GA in the brachytherapy for cervical cancer (22, 23). However, the choice of which approach to use depends largely on factors such as resource availability, experience, and practice setting.

#### Operation-Related Side Effects

Our results revealed that pain was the most common acute side effect after patients woke up from anesthesia. Frequency and scores of pain at different stages indicated that patients suffered from different degrees of pain during brachytherapy procedure, which might cause patients to move and change the position of applicator and needles, thereby affecting the clinical outcomes (15, 16). In the present study, mean pain scores for pain were highest at 4.9 ± 1.6 points during removal time, followed by 2.1 ± 1.9 points during waiting time, and we provided subcutaneous analgesia to alleviate pain when pain scores were ≥6 points. Wiebe E et al. reported that mean scores for pain were highest during waiting time, 3.3 ± 2.6 points, followed by 2.7 ± 2.1 points during removal time (24). This might be because more needles were used in the present study. Therefore, physicians should focus on the removal time and waiting time, and be prepared to treat the pain occurring during removal time and waiting time. However, most patients had mild–moderate pain during BT procedures, which was acceptable.

Our results further revealed vaginal bleeding only occurred during removal time, with more bleeding volume when more needles were applied, which was consistent with previous studies. For example, Walter (25) found neither bleeding nor other adverse reactions in 10 patients, who underwent 20 BT sessions using 66 needles. Fokdal et al. (10) reported that 4% of enrolled patients, who underwent 72 sessions of BT using 385 needles, required blood transfusion. This could be attributed to the fact that needles damaged pudendal capillaries. Our results revealed an average vaginal bleeding volume of 44.4 (SD 96.4) ml. However, Lopez-Picado et al. (26) proposed the following formula for calculating blood loss volume was as follows: Estimation of blood loss volume (EBLV) (ml) = [ETBV ICSH × (initial hematocrit − final hematocrit) + transfused red cell volume]/mean hematocrit, where ETBV ICSH is the abbreviation for Estimation of total blood volume of International Council for Standardization in Hematology (27), Women: Estimation of total blood volume of the patient (ETBV) (ml) = weight (kg)^0.425^ × height (cm)^0.725^ × 0.007184 × 2.217 + age (years) × 1.06. Based on this formula, the average EBLV was expected be 64.8 (SD 9.9) ml, which was higher than the 44.4 ml observed herein. Two reasons might contribute to the result. Firstly, patients might have been bleeding without vaginal discharge; Secondly, patients still had vaginal bleeding after removal time. Severe bleeding was a rare phenomenon during brachytherapy of cervical cancer. However, routine blood examinations before and after treatment was necessary. Therefore, we recommend that relevant departments should continuously monitor patients for detection of vital signs and check situation of vagina during removed time. Sometimes, abdominal CT scans can be performed.

In the present study, the rate of uterine perforation was 1.2% (5/407). In a related study, Sapienza et al. (28) found that the ratio of perforations in the un-guided/guided groups was 9.94 per insertion. Moreover, Onal et al. (29) suggested that MRI images taken before brachytherapy could effectively reduce the risk of uterine perforation. In their study, incidence of uterine perforation in patients who underwent MRI scanning before brachytherapy was 4% (3/67), which was significantly lower than 11% (14/133) observed in patients without MRI scanning. In the present study, only one patient with uterine perforation developed pelvic discomfort, whereas the remaining patients were asymptomatic. In addition, no symptoms appeared in these patients following antibiotic administration. Lower incidences of complete and partial uterine perforations had previously been found, with Gupta et al. (30) reporting 0.86% (37/4285) and 1.61% (69/4285), respectively. In addition, conservative treatment approaches, blood transfusion, abdominal hysterectomy/internal iliac artery ligation and partial bowel resection were used to cure the uterine perforation. Unfortunately, 12 patients with complete uterine perforation died with these deaths attributed to sepsis (eight patients) and intraperitoneal hemorrhage (four patients) resulting from intestinal injury following uterine perforation. Overall, MRI imaging before brachytherapy and the use of ultrasound-guided insertion during the operation are good for reducing incidence of uterine perforation which may prolong OTT of radiotherapy and cause fatal results.

Furthermore, we also observed hematuria, manifested as a small volume of light red urine following pulling out of the urethral catheter. Incidence of this occurrence was 1.2% (5/407). After successfully excluding the possibility of tumor invasion, as well as applicator and needle-related damaging, we concluded that urethral catheter long-term placement damaged the bladder/urethral mucosa. In a previous study, Saint et al. (31) reported a positive correlation between longer time urethral catheter stay and elevated side effects. To circumvent this problem, we strongly recommend reducing brachytherapy procedure time.

### Infection

Generally, we recorded a 6.1% (25/407) fever incidence, which was similar to 6.3% (2/32) reported by Nielsen et al. (32), and higher than 2.47% (106/4285) reported by Gupta et al. (30). On the other hand, Mendez et al. (33) found perineal infection to be the most common side effect. The low incidence might be attributed to the fact that Gupta et al. (30) used ICBT, and their patients were younger (41 years old *vs* 54 years old) than patients in the current study. Overall, these findings indicate that physicians should focus on fever and infection during BT in order to avoid prolonging OTT of radiotherapy.

### Factors Influencing Procedure Time and Acute Side Effects

Spearman correlation analysis revealed a positive association between the number of needles with the volume of vaginal bleeding and pain scores during removal time. Besides, Mann-Whitney U test revealed total procedure time with hematuria was longer than that without hematuria. Previous studies had shown that a higher number of needles result in more side effects. For example, Walter et al. (25) reported no acute adverse reactions using an average of 3.3 needles. Fokdal et al. (10) found that an average 5.4 needles caused perforation, grade 2 pain and infection in 4.2% (1/24), 16.7% (4/24), and 4.2% (1/24) of patients, respectively, whereas 4.2% (1/24) of the patients developed bleeding necessitating blood transfusion-based intervention. Moreover, Petereit DG et al. reported mean procedural time was associated with the development of an acute event (34). Overall, these results affirm that in order to effectively reduce probability of acute side effects, physicians need to reduce number of needles and procedure time.

## Conclusion

Physicians should focus on the acute side effects during brachytherapy procedure, which may prolong overall treatment time (OTT) of radiotherapy and decrease the local control rates. During BT procedure, pain and vaginal bleeding were the most common acute side effects. Mean scores for pain during removal time are highest and vaginal bleeding happen on removal time, which physicians should focus on. Shortening patients’ waiting time helps to reduce the total procedure time, thus, reduce acute side effects. While meeting the dose requirement, the number of needles should be reduced as much as possible, which is helpful to reduce acute side effects.

## Data Availability Statement

The original contributions presented in the study are included in the article/supplementary material. Further inquiries can be directed to the corresponding author.

## Author Contributions

GC conceived the design and supervised the study. JC wrote the manuscript. NZ, YL, and DH collected and analyzed the data. ZM and WY provided technical assistance with the study. All authors contributed to the article and approved the submitted version.

## Funding

This work was partially supported by grants from the National Natural Science Foundation of China [grant numbers 81201737, 31600679, 82003208, 82073331], Project of Science and Technology Department of Jilin Province [grant number 20090458], Project of Health and Family Planning Commission of Jilin Province [grant number 2014ZC054], Bethune Special Research of Science and Technology Department of Jilin Province [grant number 20160101079JC], Horizontal Project of Jilin University [grant numbers 2015373, 2016220101000686], Jilin University Technical Services Research Foundation [grant number 2015YX154], Jilin University Network Experiment Project [grant number VE2015081], Jilin University Undergraduate Education Reform Research Project [grant number 2017XYB080], Jilin University Norman Bethune Medical Department Teaching Reform Research Project [grant number B2014B137], and Project of Science and Technology Department of Jilin Province (grant number 20190303151SF).

## Conflict of Interest

The authors declare that the research was conducted in the absence of any commercial or financial relationships that could be construed as a potential conflict of interest.

## References

[1] Torre LA Bray F Siegel RL Ferlay J Lortet-Tieulent J Jemal A. Global Cancer Statistics, 2012. CA Cancer J Clin (2015) 65(2):87–108. 10.3322/caac.21262 25651787

[2] Koh W-J Abu-Rustum NR Bean S Bradley K Campos SM Cho KR. Cervical Cancer, Version 3.2019, NCCN Clinical Practice Guidelines in Oncology. J Natl Compr Canc Netw (2019) 17(1):64–84. 10.6004/jnccn.2019.0001 30659131

[3] Chargari C Deutsch E Blanchard P Gouy S Martelli H Guérin F. Brachytherapy: An Overview for Clinicians. CA Cancer J Clin (2019) 69(5):386–401. 10.3322/caac.21578 31361333

[4] Fokdal L Sturdza A Mazeron R Haie-Meder C Tan LT Gillham C. Image Guided Adaptive Brachytherapy With Combined Intracavitary and Interstitial Technique Improves the Therapeutic Ratio in Locally Advanced Cervical Cancer: Analysis From the retroEMBRACE Study. Radiother Oncol (2016) 120(3):434–40. 10.1016/j.radonc.2016.03.020 27113795

[5] Skowronek J. Current Status of Brachytherapy in Cancer Treatment - Short Overview. J Contemp Brachytherapy (2017) 9(6):581–9. 10.5114/jcb.2017.72607 PMC5808003 29441104

[6] Yoshida K Yamazaki H Kotsuma T Takenaka T Ueda MM Miyake S. Simulation Analysis of Optimized Brachytherapy for Uterine Cervical Cancer: Can We Select the Best Brachytherapy Modality Depending on Tumor Size? Brachytherapy (2016) 15(1):57–64. 10.1016/j.brachy.2015.10.002 26612700

[7] Tanderup K Fokdal LU Sturdza A Haie-Meder C Mazeron R van Limbergen E. Effect of Tumor Dose, Volume and Overall Treatment Time on Local Control After Radiochemotherapy Including MRI Guided Brachytherapy of Locally Advanced Cervical Cancer. Radiotherapy Oncol J Eur Soc Ther Radiol Oncol (2016) 120(3):441–6. 10.1016/j.radonc.2016.05.014 27350396

[8] Damato AL Lee LJ Bhagwat MS Buzurovic I Cormack RA Finucane S. Redesign of Process Map to Increase Efficiency: Reducing Procedure Time in Cervical Cancer Brachytherapy. Brachytherapy (2015) 14(4):471–80. 10.1016/j.brachy.2014.11.016 PMC4468005 25572438

[9] He MAO Zhao H Physics ZJIJoROB. The Clinic Values of the Self-made “T Type” Fixing Belt on Preventing the Utrecht Interstitial Applicator Shifts in 3D CT-Based Brachytherapy of Cervical Cancer. Int J Oncol Biol Phys (2015) 93: (3):E254–4. 10.1016/j.ijrobp.2015.07.1186

[10] Fokdal L Tanderup K Hokland SB Røhl L Pedersen EM Nielsen SK. Clinical Feasibility of Combined Intracavitary/Interstitial Brachytherapy in Locally Advanced Cervical Cancer Employing MRI With a Tandem/Ring Applicator in Situ and Virtual Preplanning of the Interstitial Component. Radiother Oncol (2013) 107(1):63–8. 10.1016/j.radonc.2013.01.010 23452917

[11] Mahantshetty U Sturdza A Naga Ch P Berger D Fortin I Motisi L. Vienna-II Ring Applicator for Distal Parametrial/Pelvic Wall Disease in Cervical Cancer Brachytherapy: An Experience From Two Institutions: Clinical Feasibility and Outcome. Radiother Oncol (2019) 141:123–9. 10.1016/j.radonc.2019.08.004 31495516

[12] Humphrey P Bennett C Cramp F. The Experiences of Women Receiving Brachytherapy for Cervical Cancer: A Systematic Literature Review. Radiogr (London Engl 1995) (2018) 24(4):396–403. 10.1016/j.radi.2018.06.002 30292512

[13] Bhanabhai H Samant R EC Grenier L Lowry S. Pain Assessment During Conscious Sedation for Cervical Cancer High-Dose-Rate Brachytherapy. Curr Oncol (2013) 20(4):e307–10. 10.3747/co.20.1404 PMC3728059 23904769

[14] Kirchheiner K Czajka-Pepl A Ponocny-Seliger E Scharbert G Wetzel L Nout RA. Posttraumatic Stress Disorder After High-Dose-Rate Brachytherapy for Cervical Cancer With 2 Fractions in 1 Application Under Spinal/Epidural Anesthesia: Incidence and Risk Factors. Int J Radiat Oncol Biol Phys (2014) 89(2):260–7. 10.1016/j.ijrobp.2014.02.018 24721589

[15] Shi D He MY Zhao ZP Wu N Zhao HF Xu ZJ. Utrecht Interstitial Applicator Shifts and DVH Parameter Changes in 3D CT-based Hdr Brachytherapy of Cervical Cancer. Asian Pac J Cancer Prev (2015) 16(9):3945–9. 10.7314/apjcp.2015.16.9.3945 25987066

[16] Pellizzon ACA. Pain Relief Procedures Before High-Dose-Rate Brachytherapy for non-Surgical Treatment of Cervix Cancer. J Contemp Brachytherapy (2018) 10(6):567–9. 10.5114/jcb.2018.81027 PMC6335560 30662480

[17] Harkenrider MM Shea SM Wood AM Chinsky B Bajaj A Mysz M. How One Institution Overcame the Challenges to Start an MRI-based Brachytherapy Program for Cervical Cancer. J Contemp Brachytherapy (2017) 9(2):177–86. 10.5114/jcb.2017.66892 PMC5437078 28533808

[18] Kim H Houser CJ Kalash R Maceil CA Palestra B Malush D. Workflow and Efficiency in MRI-based High-Dose-Rate Brachytherapy for Cervical Cancer in a High-Volume Brachytherapy Center. Brachytherapy (2018) 17(5):753–60. 10.1016/j.brachy.2018.05.001 29844009

[19] Serban M Kirisits C de Leeuw A Pötter R Jürgenliemk-Schulz I Nesvacil N. Ring Versus Ovoids and Intracavitary Versus Intracavitary-Interstitial Applicators in Cervical Cancer Brachytherapy: Results From the EMBRACE I Study. Int J Radiat Oncol Biol Phys (2020) 106(5):1052–62. 10.1016/j.ijrobp.2019.12.019 32007365

[20] Watanabe Nemoto M Nozaki-Taguchi N Togasaki G Kanazawa A Kurokawa M Harada R. New Approach to Relieving Pain and Distress During High-Dose-Rate Intracavitary Irradiation for Cervical Cancer. Brachytherapy (2015) 14(5):642–7. 10.1016/j.brachy.2015.04.009 26024785

[21] Viswanathan AN Creutzberg CL Craighead P McCormack M Toita T Narayan K. International Brachytherapy Practice Patterns: A Survey of the Gynecologic Cancer Intergroup (Gcig). Int J Radiat Oncol Biol Phys (2012) 82(1):250–5. 10.1016/j.ijrobp.2010.10.030 PMC3489266 21183288

[22] Petitt MS Ackerman RS Hanna MM Chen L Mhaskar RS Fernandez DC. Anesthetic and Analgesic Methods for Gynecologic Brachytherapy: A Meta-Analysis and Systematic Review. Brachytherapy (2020) 19(3):328–36. 10.1016/j.brachy.2020.01.006 32122807

[23] Frankart AJ Meier T Minges TL Kharofa J. Comparison of Spinal and General Anesthesia Approaches for MRI-guided Brachytherapy for Cervical Cancer. Brachytherapy (2018) 17(5):761–7. 10.1016/j.brachy.2018.05.002 29807820

[24] Wiebe E Surry K Derrah L Murray T Hammond A Yaremko B. Pain and Symptom Assessment During Multiple Fractions of Gynecologic High-Dose-Rate Brachytherapy. Brachytherapy (2011) 10(5):352–6. 10.1016/j.brachy.2011.04.001 21640664

[25] Walter F Maihöfer C Schüttrumpf L Well J Burges A Ertl-Wagner B. Combined Intracavitary and Interstitial Brachytherapy of Cervical Cancer Using the Novel Hybrid Applicator Venezia: Clinical Feasibility and Initial Results. Brachytherapy (2018) 17(5):775–81. 10.1016/j.brachy.2018.05.009 29941345

[26] Lopez-Picado A Albinarrate A Barrachina B. Determination of Perioperative Blood Loss: Accuracy or Approximation? Anesthesia analg (2017) 125(1):280–6. 10.1213/ane.0000000000001992 28368940

[27] Pearson TC Guthrie DL Simpson J Chinn S Barosi G Ferrant A. Interpretation of Measured Red Cell Mass and Plasma Volume in Adults: Expert Panel on Radionuclides of the International Council for Standardization in Haematology. Br J Haematol (1995) 89(4):748–56. 10.1111/j.1365-2141.1995.tb08411.x 7772511

[28] Sapienza LG Jhingran A Kollmeier MA Lin LL Calsavara VF Gomes MJL. Decrease in Uterine Perforations With Ultrasound Image-Guided Applicator Insertion in Intracavitary Brachytherapy for Cervical Cancer: A Systematic Review and Meta-Analysis. Gynecol Oncol (2018) 151(3):573–8. 10.1016/j.ygyno.2018.10.011 30333082

[29] Onal C Guler OC Dolek Y Erbay G. Uterine Perforation During 3-Dimensional Image-Guided Brachytherapy in Patients With Cervical Cancer: Baskent University Experience. Int J Gynecol Cancer (2014) 24(2):346–51. 10.1097/IGC.0000000000000048 24407583

[30] Gupta P Aich RK Deb AR. Acute Complications Following Intracavitary High-Dose-Rate Brachytherapy in Uterine Cancer. J Contemp Brachytherapy (2014) 6(3):276–81. 10.5114/jcb.2014.45493 PMC4200184 25337129

[31] Saint S Trautner BW Fowler KE Colozzi J Ratz D Lescinskas E. A Multicenter Study of Patient-Reported Infectious and Noninfectious Complications Associated With Indwelling Urethral Catheters. JAMA Intern Med (2018) 178(8):1078–85. 10.1001/jamainternmed.2018.2417 PMC6143107 29971436

[32] Nielsen AA Liyanage TA Leiserowitz GS Mayadev J. Optimal Perioperative Anesthesia Management for Gynecologic Interstitial Brachytherapy. J Contemp Brachytherapy (2017) 9(3):216–23. 10.5114/jcb.2017.68767 PMC5509989 28725244

[33] Mendez LC Weiss Y D’Souza D Ravi A Barbera L Leung E. Three-Dimensional-Guided Perineal-Based Interstitial Brachytherapy in Cervical Cancer: A Systematic Review of Technique, Local Control and Toxicities. Radiother Oncol (2017) 123(2):312–8. 10.1016/j.radonc.2017.03.005 28351521

[34] Petereit DG Sarkaria JN Chappell RJ. Perioperative Morbidity and Mortality of High-Dose-Rate Gynecologic Brachytherapy. Int J Radiat Oncol Biol Phys (1998) 42(5):1025–31. 10.1016/s0360-3016(98)00349-6 9869225

